# Natural Products for the Prevention and Treatment of Chronic Inflammatory Diseases: Integrating Traditional Medicine into Modern Chronic Diseases Care

**DOI:** 10.1155/2018/9837863

**Published:** 2018-04-01

**Authors:** Ji Hye Kim, Gorkem Kismali, Subash C. Gupta

**Affiliations:** ^1^Laboratory of Clinical Biology and Pharmacogenomics, Department of Preventive Medicine, College of Korean Medicine, Kyung Hee University, 26 Kyungheedae-ro, Dongdaemun-gu, Seoul 02453, Republic of Korea; ^2^Department of Biochemistry, Ankara University Faculty of Veterinary Medicine, 06110 Ankara, Turkey; ^3^Department of Biochemistry, Institute of Science, Banaras Hindu University, Varanasi 221005, India

Chronic inflammation can cause several diseases and conditions, including some cancers, obesity, asthma, rheumatoid arthritis, atherosclerosis, ischemic heart disease, and ulcerative colitis. Despite the fact that several researches were performed on prevention and treatment of inflammation-related diseases, the overall incidence has not changed significantly. Moreover, the exact mechanism underlying the process of these diseases still remains hidden. In humans, inflammation is considered a common feature of these diseases, which might be responsible for the dysfunction or dysregulation of specific cells and tissues that contribute to the origin and development of the disease. Due to the fact that chronic inflammation may play crucial roles in disease initiation, a broad spectrum of preclinical and clinical studies had been investigated to reveal the mechanisms of chronic inflammation in the biological system and to develop drugs to overcome inflammation mediated diseases.

Natural products from the herbal remedy, medicinal plants, functional foods, and their constituent have been used for the treatment of various diseases including cancer from ancient time to recent days; increasing emphasis has been focused on the research on traditional medicine: many herbs and medicinal plants. This requires new approaches to overcome inflammation mediated diseases and thus natural products could be efficacious sources for prevention and treatment of these diseases.

Since this view has made preclinical and clinical researchers start exploring the potential of natural products to overcome inflammation mediated diseases, our journal published a special issue devoted to the topic of natural products for the prevention and treatment of chronic inflammatory diseases with the integration of traditional medicine into modern chronic diseases care. The result is a collection of 17 outstanding articles submitted by investigators across countries worldwide.

C. Cheon et al. in Korea (in “Efficacy and Safety of Sipjeondaebo-Tang for Anorexia in Patients with Cancer: A Pilot, Randomized, Double-Blind, Placebo-Controlled Trial”) and S. Yuan et al. in China (in “Effectiveness and Safety of the Combination of the Traditional Chinese Medicine Prescription Jade Screen and Desloratadine in the Treatment of Chronic Urticaria: A Systematic Review and Meta-analysis of Randomized Controlled Trials”) have focused on the efficacy and safety of traditional medicines in clinical trials. These authors concluded that both* Sipjeondaebo-tang* and* Jade Screen/desloratadine* had no significant difference between treated group and placebo group in adverse reaction. However, there remains a need for further large scale studies to support these clinical applications.

From an* in vitro* research, the application of* Wannachawee* Recipe in human keratinocytes is to study its antipsoriatic activity on suppressing inflammatory cytokine production. M. N. Takuathung et al. in Thailand (in “Effects of Wannachawee Recipe with Antipsoriatic Activity on Suppressing Inflammatory Cytokine Production in HaCaT Human Keratinocytes”) have exploited the mechanisms for* Wannachawee* Recipe's antiproliferation and anti-inflammatory effects in HaCaT cells. They highlight that this is the first study to provide convincing evidence that this herbal remedy is a potential candidate for development of effective psoriasis therapies.

From* in vivo* researches on arthritis, natural products application is to identify potential biomarkers associated with adjuvant-induced arthritis and investigate the mechanism of action of those medicines. X. Wang et al. in China (in “*K* Nearest Neighbor Algorithm Coupled with Metabonomics to Study the Therapeutic Mechanism of Sendeng-4 in Adjuvant-Induced Rheumatoid Arthritis Rat”) have emphasized the importance of a serum metabolite profile analysis to identify potential biomarkers associated with adjuvant-induced arthritis to show how* Sendeng-4 *acts. L. Dong et al. in China (in “Astilbin from* Smilax glabra* Roxb. Attenuates Inflammatory Responses in Complete Freund's Adjuvant-Induced Arthritis Rats”) approached the effect of the compound* astilbin *from* Smilax glabra *Roxb. and potential mechanism on attenuation of the inflammatory response in adjuvant-induced arthritic rats.

There are potential natural products applications for fibrosis in organs. Y. Lin et al. in China (in* “*Flavanones from* Sedum sarmentosum *Bunge Alleviate CCl4-Induced Liver Fibrosis in Rats by Targeting TGF-*β*1/T*β*R/Smad Pathway In Turn Inhibiting Epithelial Mesenchymal Transition*”*) have evaluated the therapeutic effects of flavanones from* Sedum sarmentosum* Bunge on CCl_4_-induced liver fibrosis in rats and the underlying mechanisms of action. Likewise, M. Li et al. in China (in “Long-Term Effects of TCM Yangqing Kangxian Formula on Bleomycin-Induced Pulmonary Fibrosis in Rats via Regulating Nuclear Factor-*κ*B Signaling”) have examined the long-term effects of* Yangqing Kangxian formula* and evaluated the potential mechanisms in lung. Y. Tian et al. and H. Wang et al. in China (in “Bufei Yishen Granules Combined with Acupoint Sticking Therapy Suppress Inflammation in Chronic Obstructive Pulmonary Disease Rats: Via JNK/p38 Signaling Pathway” and “Xiaoqinglong Decoction Attenuates Chronic Obstructive Pulmonary Disease in Rats via Inhibition of Autophagy”) have emphasized the importance of mechanism study of two traditional Chinese medicines, widely used to treat chronic obstructive pulmonary disease (COPD).

Obesity is a metabolic disorder characterized by an excess accumulation of fat in the body. Recently, it has been widely suggested that certain gut microbiota increases metabolic endotoxin secretion, especially lipopolysaccharide (LPS) leading to chronic inflammation, resulting in obesity. Y. Liu et al. in China (in “Herbal Medicine for the Treatment of Obesity: An Overview of Scientific Evidence from 2007 to 2017”) have reviewed the possible effects and mechanisms of diverse herbal medicine in preclinical and clinical researches for the treatment of obesity. Similarly, D. Lee et al. in Korea (in “Therapeutic Effect of* Cucumis melo* L. Extract on Insulin Resistance and the Gut Microbiome in Lep^ob^/Lep^ob^ Mice”) have explored the effects of cucumins on obesity-induced insulin resistance (IR) in leptin-deficient Lep^ob^/Lep^ob^ mice.

In summary, this special issue provides an important evidence of the application of natural products in preclinical researches and clinical trials across the world. Besides what is stated, there are natural products applications for other chronic inflammation associated diseases: atherosclerosis and ischemia in the issue. Hopefully, this publication will provide a strong knowledge for therapeutic or preventative utilities of natural products in chronic inflammatory diseases.

*Ji Hye Kim* *Gorkem Kismali* *Subash C. Gupta*

